# Association between *CNR1* gene polymorphisms and susceptibility to diabetic nephropathy in Iraqi patients with T2DM

**DOI:** 10.25122/jml-2023-0181

**Published:** 2023-11

**Authors:** Raghda Hisham Aljorani, Eman Saadi Saleh, Khalaf Gata Al Mohammadawi

**Affiliations:** 1 Department of Clinical Laboratory Sciences, Faculty of Pharmacy, Al-Rafidain University College, Baghdad, Iraq; 2 Department of Clinical Laboratory Sciences, College of Pharmacy, University of Baghdad, Baghdad, Iraq; 3 Specialized Center for Endocrinology and Diabetes, Baghdad, Iraq

**Keywords:** cannabinoid receptor 1, diabetes mellitus, diabetic nephropathy, gene polymorphism

## Abstract

In individuals with type 2 diabetes mellitus (T2DM), the cannabinoid receptor 1 (*CNR1*) gene polymorphism has been linked to diabetic nephropathy (DN). Different renal disorders, including DN, have been found to alter cannabinoid (CB) receptor expression and activation. This cross-sectional study aimed to investigate the relationship between *CNR1* rs1776966256 and rs1243008337 genetic variants and the risk of developing DN in Iraqi patients with T2DM. The study included 100 patients with T2DM, divided into two groups: 50 with DN and 50 without DN. Genotyping of *CNR1* rs1776966256 and rs1243008337 polymorphisms was conducted using PCR in DN patients and control samples. The distribution of rs1776966256 and rs1243008337 genotypes and alleles between the two groups revealed statistically significant differences. The frequencies of the GG and AG genotypes of *CNR1* rs1776966256 were significantly different between DN patients and the control group. Additionally, compared to the A allele, the G allele of this polymorphism was linked to a higher incidence of DN (p=0.0001). Patients with the genetic polymorphism rs1243008337 had higher genotypes of CC and AC and were more likely to develop DN in the polymorphism genotype than the wild genotype. Additionally, compared to the A allele, the C allele was linked to a higher chance of developing DN (p=0.0001). Both rs1776966256 and rs1243008337 polymorphisms were correlated with the development of diabetic nephropathy.

## INTRODUCTION

The most severe microvascular consequence of diabetes mellitus is diabetic nephropathy (DN) [1]. As a significant public health issue, it is characterized by increased excretion of urine albumin, decreased glomerular filtration rate, or both. According to predictions, the prevalence of diabetic nephropathy will keep rising, providing a significant burden to the healthcare system and leading to higher morbidity and mortality rates [2, 3]. Endocannabinoids (ECs) are endogenous, bioactive lipid mediators that primarily function through the cannabinoid-1 (CB1) and cannabinoid-2 (CB2) receptors, which are G protein-coupled receptors. Adenyl cyclase activity may be inhibited (or, in certain circumstances, increased), and numerous mitogen-activated protein kinases (MAPKs) may be activated as part of the complicated signaling of these receptors, depending on the cell type [4, 5]. While CB1 receptors were initially thought to be predominantly expressed in the central nervous system, emerging research over the past two decades has revealed their presence and importance in various peripheral organs, including the kidneys. Both tubular epithelial cells and glomeruli express the CB1 receptor. The endothelium of intrarenal arteries contains the CB1 receptor. Under normal conditions, the EC system plays a critical role in maintaining renal homeostasis by influencing several aspects of kidney function. It regulates renal hemodynamics, modulates tubular salt reabsorption, and influences urine protein excretion [5]. The expression and function of CB receptors have been observed to change in several renal disorders, including DN [6, 7]. The main renal consequences of diabetes, such as progressive kidney diseases, are brought on by an increase in ROS generation, which eventually results in DN through various mechanisms [8, 9]. The renal cannabinoid receptors have opposing effects on oxidative stress. The CB1 receptor promotes cell death, oxidative/nitrative damage, and inflammation by activating p38-MAPK [10]. Although the pathophysiology of DN is somewhat complex, there is growing evidence that genetic variables play a role in susceptibility, suggesting that genetic factors contribute to DN risk. While numerous studies have investigated the involvement of genetic markers in the etiology of the disease, such research has not been conducted in Iraq. Single nucleotide polymorphisms (SNPs) have been discovered in a large number of genes [11], leading to a large number of gene variants that significantly contribute to the genetic susceptibility to DN [12]. The early detection and treatment of DN can greatly benefit from identifying genetic variations at the biomarker level, as this enables the identification of individuals at a higher risk of developing the disease [13]. The CB1 polypeptide, which has 473 amino acids, is encoded by the *CNR1* gene, which belongs to the family of inhibitory G protein-coupled receptors and is located on chromosome 6q14-q15 [14]. This gene exhibits various polymorphisms that have been observed in diverse population groups. Recent studies have revealed a correlation between *CNR1* gene polymorphisms and DN and associations with other clinical conditions such as metabolic syndrome [13]. To our knowledge, there have been no previous investigations into the role of SNPs within the CNR-1 gene in the pathogenesis of DN in Iraq. Therefore, the present study was designed to explore the potential existence of SNPs in CB1 receptors Rs1776966256 and rs1243008337 within a sample of Iraqi patients with T2DM and their association with the progression to DN.

## MATERIAL AND METHODS

### Patient selection and study design

We recruited a total of 100 patients with type 2 diabetes mellitus (T2DM), of both genders, who regularly attended the Al-Rusafa, Baghdad, Specialized Center for Endocrinology and Diabetes. The control group consisted of patients with diabetes without DN. T2DM diagnosis was made following the guidelines of the American Diabetes Association (ADA). Diagnosis of diabetic nephropathy was established based on comprehensive evaluations, including patient questionnaires, clinical assessments, laboratory findings, and the urinary albumin creatinine ratio (ACR) exceeding 30 mg/g. Patients with type 1 diabetes, hypertension, urethral calculi tumors (of any kind), gestational diabetes, severe cardiac, liver, and renal function failure, renal disease, and other diseases predisposing to proteinuria were excluded from the study. In addition, we excluded patients who were obese, taking nephrotoxic medications, anti-inflammatory drugs, lipid-lowering medications, and patients from different ethnic groups (Kurdish, Turkmen).

We used a questionnaire to gather data on age, gender, duration of T2DM, blood pressure, drinking and smoking habits, and previous medication use. Anthropometric measurements, including height, weight, waist circumference, and body mass index (BMI), calculated as weight divided by height squared in kg/m^2^), were recorded. Blood samples were collected to measure total cholesterol, high-density lipoprotein (HDL), low-density lipoprotein (LDL), triglycerides, estimated glomerular filtration rate (eGFR), serum creatinine, fasting blood sugar, and HbA1c levels. Random spot urine samples were collected in appropriate containers for immediate measurement of urinary albumin and creatinine levels, which were used to calculate the ACR following standard procedures.

### DNA extraction and gene genotyping

Two milliliters of venous blood specimens were collected and stored at -20°C until DNA extraction and gene genotyping. The CNR-1 gene variations were determined by polymerase chain reaction (PCR) using the Easy Pure^®^ Blood Genomic DNA Kit (Catalog No.: EE121). The extracted DNA was stored at −20°C, and DNA amplification of a single nucleotide polymorphism (SNP) was performed with primers (forward 5′-CCCTCTGCTTGCAATCATGG, reverse 5′-TGTGTAGCCAAAGGTTTCCC). The PCR conditions were set as follows: initial denaturation at 94°C for 5 min, 35 cycles of denaturation at 94°C for 30 s, annealing at 60°C for 40 s, extension-1 at 72°C for 45 sec, and final extension at 72°C for 5 min. The resulting fragments were electrophoresed on a 2% agarose gel stained with ethidium bromide in order to determine the genotypes of the patients and controls for both polymorphic sites. The PCR products and primers used in the current study underwent Sanger DNA sequencing analysis to identify any polymorphisms.

### Statistical analysis

In this study, we employed the IBM Statistical Package for the Social Sciences (SPSS) software, version 26, to explore the impact of various factors on the study parameters. The statistical analysis included one-way analysis of variance (ANOVA), t-tests for mean comparisons, and chi-square tests for percentage comparisons, with significance levels set at 0.05 and 0.01 probability. Figures for this study were created using the GraphPad Prism 9 program. Genotyping was conducted using WINPEPI and SPSS programs [15, 16].

## RESULTS

### Demographic and clinical characteristics

The demographic, anthropometric, clinical, and biochemical characteristics of all participants enrolled in this study were summarized in Table 1. There were no significant differences in age, gender, disease duration, smoking, and drinking habits between the study groups (p>0.05). On the other hand, there was a highly significant difference in the serum levels of creatinine, ACR, and eGFR between the control group and the patient groups (p<0.05). ACR and creatinine levels were higher in the DN patient group than in the control group, while eGFR showed the lowest levels in the DN group. When compared between both groups, the mean serum levels of HbA1c, FBG, and BMI revealed a non-significant difference (p>0.05). The distribution of DN patients according to the KIDGO eGFR stages was significantly different between the groups, with the majority of controls at stage 1 and only 10 at stage 2. In contrast, the distribution of DN patients was uneven, with most patients at stage 1 (Table 1).

**Table 1:** Sociodemographic characteristics of the study groups

Characteristic		Group 1 (DN) n=50	Group 2 (T2DM) n= 50	p-value
Age (year)		50.92±9.23	52.62±10.32	0.3
Gender	Female	22 (44%)	26 (42%)	0.4
	Male	28 (56%)	24 (48%)	
BMI (kg/m^2^)		26.76±3.07	26.52±3.16	0.7
Smoking	Smoker	11 (22%)	13 (26%)	0.6
	Nonsmoker	39 (78%)	37 (74%)	
Alcohol consumption	Yes	5 (10%)	3 (6%)	0.2
	No	45 (90%)	47 (94%)	
T2DM duration (year)		8.7±4.33	8.08±4.3	0.4
HbA1c (%)		8.89±2.32	8.13±2.30	0.1
FSG (mmol/L)		9.84±2.44	8.95±2.12	0.3
ACR 3-30 (mg/g)		85.33±66.80	13.12±6.92	0.0001**
Unit of S. Creatinine (µmol/L)		73.48±26.89	58.04±16.95	0.001**
eGFR		98.16±31.49	123.92±49.90	0.003**
CKD stages (according to eGFR ml/min/1.73m^2^				
Stage 1 (≥90)		31	40	0.01**
Stage 2 (60-90)		12	10	
Stage 3 (30-60)		7	0	
Stage 4 (15-29)		0	0	
Stage 5 (<15)		0	0	

Group 1: T2DM patient with DN, Group 2: control group T2DM patient without DN. BMI: body mass index, T2DM: type 2 diabetes mellitus, HbA1c: glycated hemoglobin, FBS: fasting blood sugar, eGFR: estimated glomerular filtration rate, ACR: urinary albumin-creatinine ratio, CKD: chronic kidney disease; Continuous variable expressed as median ±SD; *: significant difference between groups.

### Genetic analysis

**Figure 1:**
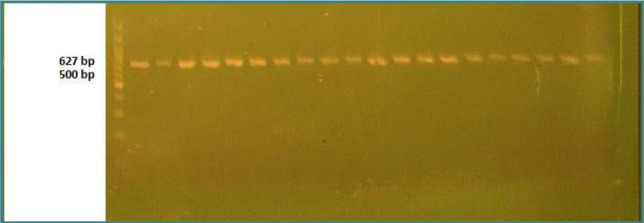
Electrophoresis of a 627 bp fragment of human CNR1 gene on ethidium bromide-stained agarose (1.5%) gel at 45 volt/cm^2^ and 1× TBE buffer for one hour

**Figure 2:**
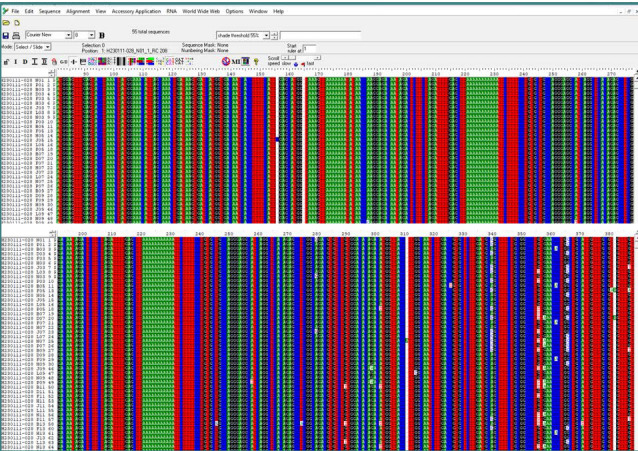
The BioEdit program sequence alignment findings for the Homo sapiens cannabinoid receptor 1 (CNR1) transcript variant 10 fragments confirmed the compatibility of sample sequences with a reference sequence from the Gene Bank

**Figure 3:**
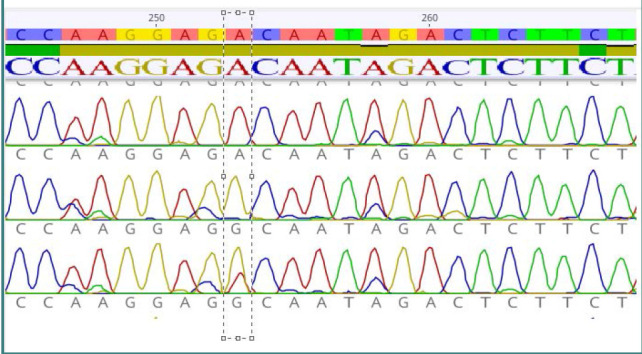
Analysis of rs1776966256 SNP of *CNR1* gene

**Figure 4:**
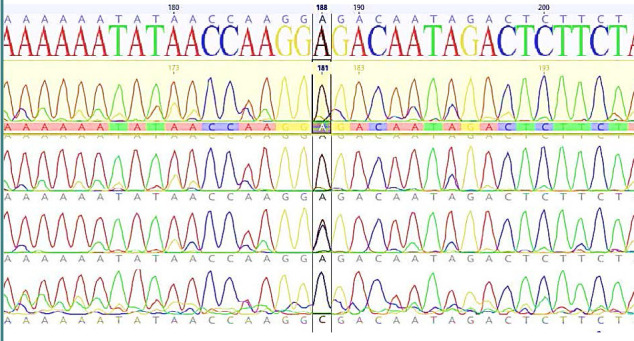
Analysis of rs1243008337 SNP of *CNR1* gene

All DNA samples from patients and controls underwent DNA sequencing for the *CNR1* gene (Figure 1). The results of the *CNR1* gene sequencing revealed the absence of the G1359A variant (rs1049353). However, two other SNPs, namely rs1776966256 and rs1243008337, were detected and subsequently subjected to further analysis, as shown in Figure 2. The results of the analysis of the rs1776966256 SNP of the *CNR1* gene using Sanger sequencing are presented in Figure 3. A single "A" peak indicates an A homozygous genotype, while a single "G" peak indicates a G homozygous genotype. The presence of both "A" and "G" peaks indicates an A/G heterozygous genotype. Similarly, Figure 4 illustrates the Sanger analysis of the rs1243008337 SNP of the *CNR1* gene, where a single "C" peak signifies a C homozygous allele, and the presence of both "C" and "A" peaks indicates a C/A heterozygous allele.

### Association with diabetic nephropathy

The association between *CNR1* gene polymorphisms and the risk of diabetic nephropathy in different genetic models among Iraqi patients with type 2 diabetes is summarized in Table 2. The data analysis revealed significant differences in the GG and AG genotypes frequencies of *CNR1* rs1776966256 between patients with DN and control subjects (p=0.008, p=0.006). Moreover, the G allele of this polymorphism was associated with an increased risk of diabetic nephropathy compared to the A allele (p=0.0001) (OR=4.33; 95% CI= 2.31 to 8.19). Furthermore, patients with rs1243008337 genetic polymorphism exhibited significantly higher frequencies of the CC and AC genotypes and were more likely to develop DN (OR=5.27; 95% CI =1.16-36.99; p=0.02 and OR=5.52; 95% CI=2.31-13.22; p=0.0001) compared to the wild genotype. The C allele of this polymorphism was associated with an increased risk of diabetic nephropathy compared to the A allele (p=0.0001) with an OR of 5.08 (95% CI= 2.65 to 9.88).

**Table 2:** Genotypes and allele frequency of rs1776966256 and rs1243008337 in DN patients and controls

Genotype/Allele	rs1776966256			
	Control group (n=50)	DN group (n=50)	p-value	OR 95% CI
AA	32 (64.00%)	9 (18.00%)	-----	1.00 reference
AG	16 (32.00%)	30 (60.00%)	0.006*	3.19 (1.39 to 7.31)
GG	2 (4.00%)	11 (22.00%)	0.008*	6.77 (1.54 to 46.49)
A	80 (80.00%)	48 (48.00%)	----	1.00 reference
G	20 (20.00%)	52 (52.00%)	0.0001*	4.33 (2.31 to 8.19)
	**rs1243008337**			
AA	35 (70.00%)	8 (16.00%)	-------	1.00 reference
AC	13 (26.00%)	33 (66.00%)	0.0001*	5.52 (2.31 to 13.22)
CC	2 (4.00%)	9 (18.00%)	0.02*	5.27 (1.16 to 36.99)
A	83 (83.00%)	49 (49.00%)	------	1.00 reference
C	17 (17.00%)	51 (51.00%)	0.0001*	5.08 (2.65 to 9.88)

* Significant difference between groups; rs:- reference SNP, OR: odd ratio, CI, confidence interval

### Hardy-Weinberg Equilibrium

For rs1776966256 SNP, the observed genotype frequencies exhibited no significant differences from those predicted, with a frequency of 0.36 in patients and 1.00 in controls, indicating conformity to the Hardy-Weinberg Equilibrium (HWE). The genotype AG appeared to be the most common genotype in the Iraqi population, accounting for a substantial portion, with frequencies of 46. The remaining genotypes, GG and AA, were observed at frequencies 13 and 41, respectively, as shown in Table 3. Similarly, the analysis of HWE for rs1243008337 indicated no significant differences between the observed genotype frequencies and those predicted, both in patients and controls. In patients, the HWE value was 0.07, while in controls, it was 0.8 (Table 4).

**Table 3:** Hardy Weinberg Equilibrium of rs1776966256 in patients and control

Groups rs1776966256		AA	AG	GG	χ^2^	p-value
Patients Genotype	Observed no.	9	30	11	2.04	0.36 N.S
	Expected no.	11.52	24.96	13.52		
Control Genotype	Observed no.	32	16	2	0	1.00 N.S
	Expected no.	32	16	2		
Total Observed		41	46	13		

**Table 4:** Hardy Weinberg Equilibrium of rs1243008337 in patients and control

Groups rs1243008337		AA	AC	CC	χ^2^	p-value
Patients Genotype	Observed no.	8	33	9	5.14	0.07 N.S
	Expected no.	12.005	24.99	13.005		
Control Genotype	Observed no.	35	13	2	0.31	0.8 N.S
	Expected no.	34.445	14.11	1.445		
Total Observed		43	46	11		

### Glycemic and renal parameters

Analysis of fasting serum glucose (FSG) and glycated hemoglobin (HbA1c) in both patient and control groups using one-way ANOVA showed no significant effect of the rs1776966256 variant on glycemic index (Table 5). There were no significant differences in serum creatinine, eGFR, and ACR between the wild and the polymorphism genotype, as shown in Table 5. A similar result was observed for the rs1243008337 variant (Table 6), where there was no significant effect on HbA1c, FSG, serum creatinine, eGFR, or ACR between the patient and control groups.

**Table 5:** Association between rs1776966256 with HbA1c, FSG, serum creatinine, estimated glomerular filtration rate, and albumin/creatinine ratio

Groups	rs1776966256		HbA1c	FSG	S. creatinine	eGFR	ACR
DN	AA	Mean	8.08±2.33	11.36±5.36	69.66±13.69	96.06±17.62	87.17±53.99
	AG	Mean	9.02±2.15	10.06±4.37	71.00±27.80	102.40±34.58	71.03±51.09
	GG	Mean	9.21±2.78	8.01±3.52	83.36±31.81	88.30±31.27	122.82±99.01
	p-value		0.5	0.2	0.3	0.4	0.08
Control	AA	Mean	8.29±2.46	8.79±5.59	58.40±18.77	129.20±56.80	12.32±6.81
	AG	Mean	7.88±1.95	9.24±4.51	55.56±13.01	116.97±34.99	14.64±7.46
	GG	Mean	7.45±3.32	9.30±2.82	72.00±11.31	95.15±17.04	13.76±3.56
	p-value		0.7	0.9	0.4	0.5	0.5

Data are presented as mean±SD, FSG: fasting serum glucose, HbA1c glycated hemoglobin

**Table 6:** Association between rs1243008337 and HbA1c, FSG, serum creatinine, estimated glomerular filtration rate, albumin/creatinine ratio

Groups	rs1243008337		HbA1c	FSG	S. creatinine	eGFR	ACR
DN	AA	Mean	8.25±2.52	10.56±5.82	70.50±14.55	93.90±16.58	72.92±51.21
	AC	Mean	9.14±2.23	10.39±4.21	69.60±26.93	104.44±33.80	76.11±51.22
	CC	Mean	8.54±2.55	7.21±3.26	90.33±30.86	78.91±25.77	130.16±108.53
	p-value		0.5	0.1	0.l	0.08	0.08
Control	AA	Mean	8.20±2.49	8.82±5.53	59.78±18.56	125.13±55.27	12.54±6.69
	AC	Mean	7.51±1.76	8.40±4.23	54.69±14.95	125.40±43.65	14.11±7.28
	CC	Mean	10.82±1.58	11.75±6.57	59.00±.00	106.25±20.43	12.61±13.49
	p-value		0.1	0.7	0.6	0.8	0.7

Data are presented as mean±SD

## DISCUSSION

End-stage kidney disease, often attributed to diabetic nephropathy, is a frequent and serious complication of diabetes that shortens life expectancy and is still not adequately treated [17, 18]. While hyperglycemia is recognized as a primary cause of glomerulopathy in DN, progressive kidney damage in patients with diabetes with good glucose control suggests the involvement of additional contributing factors [19]. Determining other factors that may influence the progression of DN is also crucial, such as the potential role of the cannabinoid type 1 receptor gene polymorphism in type 2 diabetes with nephropathy. A prior investigation in this area indicated that genetic and environmental variables can cause DN [14]. According to earlier research, hyperglycemia and increased renin-angiotensin system (RAS) activity are the two main pathogenic factors that cause the endocannabinoid/cannabinoid1 receptor (CB1R) system to become overactive in podocytes, which in turn contributes to the development of diabetes and its complications [5, 20] and has detrimental effects on the body [20]. Our results support the findings of Buraczynska *et al*., underscoring the connection between *CB1R* and diabetic nephropathy, as shown by the association between nephropathy in T2DM patients and a polymorphism in the *CNR1* gene [21]. Therefore, *CNR1* gene polymorphisms may influence *CB1R* signaling in podocytes and result in CB1 receptor activation either directly or indirectly (through its metabolic ramifications) by enhancing diabetes-related inflammation and ROS generation, encouraging tissue injury, and promoting the emergence of diabetic complications.

In the current study, the genetic relationship between the *CNR1* rs1776966256 and rs1243008337 polymorphisms and susceptibility to diabetic nephropathy in the Iraqi population was investigated. The distribution of rs1776966256 and rs1243008337 genotypes and alleles between the analyzed groups showed a statistically significant difference. Individuals with the *CNR1* rs1776966256 polymorphism with the GG and AG genotypes had a higher risk of developing DN. Additionally, compared to the A allele, the mutant homozygote G allele of this polymorphism was linked to a higher incidence of diabetic nephropathy. We found that patients with CC or AC genotypes were considerably more likely to develop DN than wild genotypes for the rs1243008337 polymorphism. Additionally, compared to the A allele, the C allele of this polymorphism was linked to a higher risk of diabetic nephropathy.

This study presents a novel result in a Middle Eastern/Iraqi population in the province of Baghdad, although the mutant homozygote CC was not significant due to its low-frequency variant and the limited sample size. Interestingly, no prior research has been done on these SNPs. Recent genome-wide association studies have been undertaken to investigate the relationship between T2DM and nephropathy [13, 21]. However, no correlation between the *CNR1* variants rs1243008337 and rs1776966256 and the presence of diabetic nephropathy has been found. Despite the success of genome-wide association studies as a potent tool in the analysis of complex diseases, identifying all genetic risk factors associated with the disease is challenging due to its complexity. It is unclear how the *CNR1* gene polymorphism affects the transmission of vulnerability to diabetic nephropathy. *CNR1* may affect a number of metabolic processes, and individual differences in the metabolic profile, including glycemic control, dietary preferences, treatment response, obesity, blood pressure, and other related factors, may have contributed to the potential impact of *CNR1* polymorphisms on the likelihood of developing diabetic nephropathy.

This study has limitations, including a small sample size and a narrow focus on a single facility in Baghdad. Therefore, care must be taken when extrapolating the findings of this study to the entire nation. Additionally, interactions among different risk alleles, environmental factors, adherence to treatment, and dietary practices can influence the presentation and outcomes of diabetes. Due to the complex interplay of these factors, the significance of a studied polymorphism in determining the phenotype may be either overestimated or underestimated.

## CONCLUSION

There was a predictable association between *CNR1* polymorphisms (rs1776966256 and rs1243008337) and susceptibility to diabetic nephropathy in the Iraqi population. This finding provides valuable insights into the genetic factors influencing this condition and should be confirmed in a larger replication study.

## Data Availability

The data of this study is available by request.

## References

[1] Salih BH, Ali SH, Allehibi KI (2019). Serum aldosterone levels in patients with diabetic nephropathy in relation to vascular calcification. Iraqi J Pharm Sci.

[2] Hussein AM, Ali SH, Rahma AM, Al-Shami AA (2010). Anemia in diabetic patients without microalbuminuria; in relation to type of diabetes, glycemic control, and hs-CRP levels. Al-Nahrain J Sci.

[3] Salman O, Merdaw MA, Almaliky AA (2022). A Novel single nucleotide polymorphism of interleukin-10 gene is linked to type 2 diabetes mellitus in Iraqi patients with toxoplasmosis. Iraqi J Pharm Sci.

[4] Crasto W, Patel V, Davies MJ, Khunti K (2021). Prevention of microvascular complications of diabetes. Endocrinol Metab Clin.

[5] Horváth B, Mukhopadhyay P, Haskó G, Pacher P (2012). The endocannabinoid system and plant-derived cannabinoids in diabetes and diabetic complications. Am J Pathol.

[6] Tam J (2016). The emerging role of the endocannabinoid system in the pathogenesis and treatment of kidney diseases. J Basic Clin Physiol Pharmacol.

[7] Ameen IA, Saleh ES, Mhaibes SH, Taha KN (2020). Evaluation of some inflammatory cytokines and glycated hemoglobin in uncontrolled type 2 diabetes mellitus with nephropathy. Indian J Forens Med Toxicol.

[8] Mohammed MM, Hadi RA, Salman IN (2022). Potential positive effects using coenzyme Q10 Supplement as adjuvant therapy to gabapentin for managing diabetic neuropathy. Iraqi J Pharm Sci.

[9] Mahmood AR (2016). Estimation of oxidative stress and some trace elements in Iraqi men patients with type 2 diabetes mellitus. Iraqi J Pharm Sci.

[10] Mukhopadhyay P, Pan H, Rajesh M, Bátkai S (2010). CB1 cannabinoid receptors promote oxidative/nitrosative stress, inflammation and cell death in a murine nephropathy model. Br J Pharmacol.

[11] Saleh Khudhur S, Saadi Saleh E, Hadi Alosami M, Shareef LG (2022). Association between polymorphisms within the gene coding for tumor necrosis factor (TNF)-alpha with outcomes of treatment in a sample of Iraqi patients with ankylosing spondylitis taking etanercept: an observational study. F1000Research.

[12] Pucci M, Zaplatic E, Micioni Di Bonaventura MV (2021). On the Role of Central Type-1 Cannabinoid Receptor Gene Regulation in Food Intake and Eating Behaviors. Int J Mol Sci.

[13] Zhang X, Zhu H, Xing X, Zhang C (2020). Association between cannabinoid receptor-1 gene polymorphism and the risk of diabetic nephropathy among patients with type 2 diabetes mellitus. Pharmgenomics Pers Med.

[14] Rizvi S, Raza ST, Mahdi F (2014). Association of genetic variants with diabetic nephropathy. World J Diabetes.

[15] Glover T, Mitchell K (2008). An introduction to Biostatistics.

[16] Forthofer RN, Lee ES (2014). Introduction to Biostatistics: A guide to design, analysis, and discovery.

[17] Ali SA, ALsharifi ZAR, Allawi AAM (2020). The impact of methylenetetrahydrofolate reductase gene polymorphisms on Iraqi patients with diabetic nephropathy. Int J Psychosocial Rehabil.

[18] Salih RKM, Al-Gharawi AM, Al-Lehibi KI (2012). The correlation between hyperglycemia and rheumatoid factor in type 2 diabetic patients in Al-Risafa area, Baghdad. Iraqi J Pharm Sci.

[19] Wu T, Ding L, Andoh V, Zhang J, Chen L (2023). The mechanism of hyperglycemia-induced renal cell injury in diabetic nephropathy disease: An update. Life (Basel Switzerland.

[20] Jourdan T, Szanda G, Rosenberg AZ, Tam J (2014). Overactive cannabinoid 1 receptor in podocytes drives type 2 diabetic nephropathy. Proc Natl Acad Sci U S A.

[21] Buraczynska M, Wacinski P, Zukowski P, Dragan M, Ksiazek A (2014). Common polymorphism in the cannabinoid type 1 receptor gene (CNR1) is associated with microvascular complications in type 2 diabetes. J Diabetes Complications.

